# Executive Functions in Aging: An Experimental and Computational Study of the Wisconsin Card Sorting and Brixton Spatial Anticipation Tests

**DOI:** 10.1080/0361073X.2021.1932202

**Published:** 2021-08-16

**Authors:** Andrea Caso, R. P. Cooper

## Abstract

In order to explore the effect of normal aging on executive function, we tested 25 younger adults and 25 neurologically healthy older adults on the Wisconsin Card Sorting Test (WCST) and the Brixton Spatial Anticipation Test (BRXT), two classic tests of executive function. We found that older participants were more likely than younger participants to err on both tasks, but the additional errors of older participants tended to be related to task set maintenance and rule inference rather than perseveration. We further found that the tendency to perseverate (across all participants) on the WCST was related to the tendency to produce stimulus or response perseverations on the BRXT, rather than any tendency to perseverate on BRXT rule application. Finally, on both tasks, older participants were also slower, particularly on trials following an error, than younger participants. To explore the neurocomputational basis for the observed behaviours we then extended an existing model of schema-modulated action selection on the WCST to the BRXT. We argue on the basis of the model that the performance of older participants on both tasks reflects a slower update of schema thresholds within the basal ganglia, coupled with a decrease in sensitivity to feedback.

The efficiency of executive functioning – the ability to modulate over-learned behaviors in the service of complex tasks and higher-order goals – is known to decline with age. For example, Pettigrew & Martin (2014) found that older adults (aged 64 to 87) were more susceptible to interference in a range of tasks (such as the Stroop task and a flanker task) than young adults (aged 18 to 32), while when comparing participants aged 64–87 with younger participants, Treitz, Heyder, & Daum (2007) found specific deficits in the inhibition of prepotent responses and the ability to divide attention between tasks in the older group. Many other studies, including some reviewed below, have found broadly similar effects, and some authors have even argued that cognitive performance of executive tasks in older populations can be compared to the neuropsychological profile of patients with mild frontal damage (Greenwood, 2000).

However, although there is consensus on their specific cognitive vulnerability in aging, it is unclear how the pattern of declining efficiency of these function unfolds during normal aging (Jurado & Rosselli, 2007). Moreover, while there is clear evidence of neurobiological changes with increasing age (decreased prefrontal gray matter volume: Raz et al. 1997); decreased concentration of dopamine, acetylcholine, and norepinephrine: Jacques, Ebinger, & Vauquelin 1990), the link between neurobiological changes and declining efficiency of the cognitive-level constructs involved in executive functioning is less well developed (but see Chao & Knight, 1997; Persson et al., 2006). This paper uses experimental and neurocomputational methods to investigate how potential neurobiological changes with age result in changes in executive function.

We focus on executive functions underlying performance in two related rule-induction tasks – the Wisconsin Card Sorting Test (WCST; Milner, 1963) and the Brixton Spatial Anticipation Test (BRXT; Burgess & Shallice, 1996). Both tests involve presenting participants with multiple stimuli in sequence, and require participants to induce a rule or regularity that: a) holds over the stimuli of the sequence; but b) periodically changes. Participants must do this using feedback provided after each stimulus. The tests are described in more detail below, but for current purposes it is sufficient to note that the former is widely used in the clinical assessment of executive function deficits, while the latter was designed to mimic certain aspects of the WCST while reducing the susceptibility of behavior to perceptually salient stimulus features (specifically, the color, number, and shape of a stimulus – the distinguishing features of WCST stimuli).

It is generally accepted that both the WCST and the BRXT tap a variety of executive functions, including set switching, and response inhibition, as well as processes involved in task-set maintenance and rule induction (e.g., Esther et al., 2009; Miyake et al., 2000; Reverberi, Lavaroni, Gigli, Skrap, & Shallice, 2005; Stuss et al., 2000). Moreover, at a broad level, both tasks are also known to be sensitive to age (e.g., Bielak, Mansueti, Strauss, & Dixon, 2006; Haaland, Vranes, Goodwin, & Garry, 1987), in that in both tasks older participants generally make fewer correct responses than younger participants. However, given the similarities and differences between the tests, fine grained analysis of participant performance on them offers the prospect of shedding light on whether (or how) age affects those processes required in performance of both tasks or those involved in performance of one or the other task.

Analyses of behavior on tasks such as the WCST and BRXT can also help to formulate or validate cognitive/information processing level theories of task performance, but as argued by Marr (1982), implementational level theories (or neurocomputational models, as considered here) further allow such cognitive-level theories to be linked with the neurobiological level. For example, theories that propose that behavior is the product of selection between competing schemas (as adopted here) may be further constrained with neurobiological data by supplementing such theories with computational accounts that associate the cognitive constructs of schemas and competition resolution with, for example, corticostriatal circuits, together with computational accounts of corticostriatal/basal ganglia function and dysfunction.

In the light of this, the current paper reports a study in which younger and older participants completed both the WCST and the BRXT. We find both similarities and differences in the behavior of the two participant groups on the tests. We then demonstrate how an existing neurobiological model of the modulation of schema-driven behavior can be applied to model each task, and consider how effects of aging may be incorporated into the model. More specifically, we demonstrate first that the behavior of older participants on each task may be simulated by reducing the values of two learning parameters (which we associate with cortical and subcortical learning) from the values associated with younger participant behavior, and second that within the model different values of the learning parameters can, to a certain extent, be compensated for by different levels of executive control.

## Experiment

### Introduction

As noted, our interest here is in two specific executive function tasks: the Wisconsin Card Sorting Test (WCST) and the Brixton Spatial Anticipation Test (BRXT).

The WCST requires that a participant sort a series of cards according to criteria that a) must be inferred from feedback after each trial by the participant, and that b) change without warning after a series of correct responses. The test is generally held to measure some level of executive functioning and is frequently used to assess executive (dys)function in clinical populations such as frontal patients as well as in healthy elderly participants (Heaton, 1981). Participants can commit several distinct kinds of error on the task, two of which are of particular interest with respect to executive functioning: perseverative errors (PE) and set loss errors (SL). These two forms of error are mutually exclusive, in that perseverative errors are scored when a participant continues to apply a rule that is defunct, while set loss errors are scored when the participant switches rules (despite positive feedback suggesting that the rule applied on the previous trial is correct). Consistent with some previous research (e.g., the electrophysiological study of Lange et al., 2016), we argue that these two error types depend on partially separable cognitive processes, the first of which is primarily a function of subcortical processing while the second is primarily cortical in nature. This position is also consistent with neuropsychological studies that have found different patient groups (as defined by lesion location) to be susceptible to different error types (most notably Stuss et al., 2000).

A first necessary (but not sufficient) step to argue in support of this dissociation is to show that the error types are independent, at least in some populations. An aging population is ideal, because functional decline of cognitive control in the elderly covaries with the degree of overlap in task representation (Mayr, 2001), which presumably is high across the possible rules involved in the WCST. In this regard, prior research has also found that in older populations there is an overall decline of proactive control (Paxton, Barch, Racine, & Braver, 2007; Pettigrew & Martin, 2014), defined as the ability to sustain goal-relevant information. Within the WCST, proactive control is required to maintain task set (i.e., to avoid set loss errors).

Reactive control, that is the ability to mobilize resources once interference or error is detected, is instead thought to be spared by aging. In the WCST, reactive control is required to avoid perseverative errors. Yet the findings concerning the relation between perseveration and aging are mixed. Heaton (1981) reported that individuals over 60 produce more perseverative errors than younger controls. However, Boone, Ghaffarian, Lesser, Hill-Gutierrez, & Berman (1993) failed to replicate this effect, reporting that individual older than 70 did not, and Haaland et al. (1987) found that increased perseveration appears only after the age of 80. A potential reconciliation of these findings has been offered by Rhodes (2004), who argued that age-related perseveration is moderated by the number of years of education, with more educated participants tending to commit fewer perseverative errors. Consistent with this, and with the potential dissociation of perseverative and set loss errors, Plumet, Gil, & Gaonac’h (2005) found that women over 70 with fewer than 12 years of education committed more perseverative errors than similarly aged participants with more education, but set loss errors were higher in older participants irrespective of educational level.

For current purposes, a critical element of the BRXT is that, like the WCST, it affords two distinct error types – perseverative errors and non-perseverative errors. In the standard version of the BRXT, participants are presented with a series of cards showing 10 disks, with the disks arranged in two rows of five columns, and numbered 1 to 10. One disk on each card is shaded black. The position of the shaded disk changes from one card to the next, and the participant’s task is to predict where s/he suspects the shaded disk will appear on the next card, given its location on the previous card(s). The shaded locations follow various simple temporal rules (e.g., alternating within a column, or moving left-to-right along the upper row and then right-to-left along the lower row, etc.). Burgess & Shallice (1996) classify errors on the test (i.e., incorrect predictions) into three types: perseverative errors, application of an incorrect rule, and participant responses that “are unconstrained by their previous history of performance on the task” (p. 247). Within the category of perseverative errors, Burgess & Shallice (1996) further distinguish between perseveration of the previous response, perseveration of the previous stimulus, and perseveration of the previously applied rule.

As noted, previous studies have shown that age is a predictor of errors on the BRXT. Thus, in a large norming study with participants aged 53 to 90, Bielak et al. (2006) found a strong positive correlation between total errors on the BRXT and age (r=.34, p<.01), but they did not decompose error score into the various error types. The authors also found correlations between total error score on the BRXT and performance on other tests of executive function. In particular, in addition to the BRXT, their participants completed the Hayling sentence completion test (a timed test requiring participants to generate both likely and unlikely completions to sentence frames; Burgess & Shallice, 1997) and tests of crystallized and fluid intelligence. BRXT total error score was found to correlate positively with age and negatively with education (with both effects being independently significant), but also positively with time to complete each part of the Hayling test, and negatively with the measure of fluid intelligence. For present purposes, however, and despite its suggestive nature, the theoretical import of the Bielak et al. (2006) study is unclear as it does not establish whether the increasing tendencies with age and education to err on the BRXT reflects an increase in perseverative or non-perseverative errors.

While the BRXT evidence relating to age and specific error types is unclear, the WCST evidence suggests that while set loss errors (and attentional failure errors more generally) may be a hallmark of aging, perseveration appears to be dependent on other factors that are potentially less clearly connected with the underlying neurobiology of the frontal cortex alone (such as level of education). This position is also consistent with the possibility that perseverative errors arise from an inability or reluctance to use feedback to update task representations – an inability that might be dependent on the failure to generate or apply an efficient strategy (which might in turn be dependent upon education) rather than one that is solely dependent on unspecified frontal dysfunction.

In order to examine whether, and if so how, performance on the WCST and BRXT changes with age, we therefore asked 25 younger and 25 older participants to complete a computerized version of each task (as described below). We hypothesized that in WCST we would observe significantly more set loss errors in the behavior of the older group compared to the younger group, but that there would be no significant difference in the number of perseverative errors produced by the two groups. With regard to the BRXT, we hypothesized an increase in non-perseverative errors in the older group compared to the younger group (meaning that only the total error score would be significantly greater for older participants). Furthermore, we hypothesized a positive correlation between perseverative errors in the WCST and the BRXT (and specifically perseverative rule application errors in the BRXT), on the assumption that both types of error result from suboptimal functioning of the same underlying mechanisms.

### Method

#### Participants

Participants consisted of 25 younger adults (9 men and 16 women) and 25 older adults (8 men and 17 women). The age of younger participants ranged from 19 to 53 years (M=27.1, SD=9.1). The age of older participants ranged from 62 to 84 years (M=70.8, SD=6.4). A chi-square test found no relationship between gender and age group ($\chi^{2}\left(1\right)=0.089$, p=.765). Younger participants were recruited mainly through the university participant database while older participants were recruited via charities for the elderly such as Age UK and the University of the Third Age, in London (UK). All participants were required to be free of any neurological or psychiatric diagnosis, although these conditions were not formally assessed.

#### Materials and measures

##### The Wisconsin Card Sorting Test (WCST)

We used a version of the Wisconsin Card Sorting Test in which participants were presented with a touch screen tablet computer showing four “target” cards arranged from left to right across the upper half of the screen, and a “test” card centered in the lower half of the screen. The images on the target cards showed, from left to right, one red triangle, two green stars, three yellow crosses, and four blue circles. The image on the test card varied in number, color, and shape across trials. Participants were instructed that the test card could be matched to one or more target cards according to the number, color, or shape of figures on the cards (i.e., they were informed of the potential sorting rules), and that their task was to determine the rule being followed by the computer by dragging the test card to beneath one of the four target cards, whereupon feedback (“Correct” or “Incorrect”) would be given both on screen and auditorily. Once the test card was released it remained visible on screen for one second before it disappeared. The complete task required participants to sort 64 cards (i.e., all combinations of the four numbers, colors, and shapes), with the computer’s sorting rule changing after every run of six correct responses.

The version of the WCST administered here thus differed from the standard clinical one in two ways. First, explicit instruction on the possible rules was given (as, e.g., in the 64A condition of Stuss et al., 2000). Second, each test card was removed from view once it had been sorted. The latter was included both to prevent participants from using the position of last test card as a mnemonic for the current rule, and to prevent participants who might do this from confusing the last test card with one of the earlier test cards and thereby inferring the wrong rule from cues present on the screen.

Responses were registered by the tablet in order to compute several performance measures, including: the number of categories achieved (CA), the number of total errors (TE), the number of perseverative errors (PE), and the number of set loss errors (SL).

The number of categories achieved (CA) was the number of rules correctly inferred by the participant (i.e., the number of times the participant was correct six times in succession). With 64 cards, CA could in principal range from 0 to 10. The number of total errors (TE) was the total number of times negative feedback was given. The maximum score was 64.

Perseverative errors were calculated as indicated in the manual of Heaton (1981). Thus, each response that would have been correct according to the previous sorting rule (or “set”) was counted as a PE. Moreover, if a participant selected an incorrect rule consistently and unambiguously on more than three successive trials, then immediate subsequent errors were considered to be perseverative. Set loss errors were calculated as in Stuss et al. (2000): as errors following three correct and unambiguous responses (hence we refer to this as $SL_{3}$, in contrast to Heaton, 1981, where five correct and unambiguous responses are required to establish a set). All errors after a set loss error were not counted as such. Perseverative errors and set loss errors are mutually exclusive, as the former occur following a change in the target sorting rule, while the latter occur in the absence of a change in the target sorting rule, but other errors are also possible, so TE may be more than the sum of PE and $SL_{3}$.

##### The Brixton Spatial Anticipation Test (BRXT)

We also used a touch-screen-based variant of the Brixton Spatial Anticipation Test devised by Burgess & Shallice (1996). In our version of the task, participants were presented with a set of nine small disks arranged in a circular fashion around a central point. One of the disks was always filled (i.e., it was a black). Participants were asked to touch the location on the screen where they believed the next filled disk would appear. The filled disk moved after each touch following a series of five sequential rules (e.g., move counter-clockwise), with each rule comprising ten successive disk locations. We did not disallow selection of locations outside of the nine disks, but we carefully instructed participants that targets could only occur in the nine disk locations and whenever they tapped outside of those locations text was shown on screen reminding participants to tap on one of the nine disks. Responses were registered by the device in order to compute correct and incorrect predictions, as well as response times.

Our version of the BRXT differed from that of Burgess & Shallice (1996) in the spatial arrangement of the small disks – in Burgess & Shallice (1996) 10 disks were arranged in a two-by-five rectangle – and in that we recorded response times.

Beyond response times, four measures were calculated from the series of responses. The number of Total Errors (TE) was the number of incorrect predictions (including responses where the selected location was not one of the nine disks). This could range from 0 to 50. Stimulus perseverative errors ($P_{STIM}$) were counted whenever a participant selected the current disk (which was also the previous target). Response perseverative errors ($P_{RESP}$) were scored whenever a participant selected the disk corresponding to their previous response. Note that in some circumstances, a $P_{RESP}$ error could also count as $P_{STIM}$ error. Finally, perseverative rule errors ($P_{RULE}$) were counted whenever a participant selected the response that would have been correct under the previously active rule. Since the participant was unaware of when the rule would change, some $P_{RULE}$ errors were inevitable whenever the rule changed.

#### Procedure

The study took place in an acoustically isolated booth, with each participant tested separately. All 50 participants completed both the WCST and the BRXT, with task order randomized by participant. In order to minimize distractions, participants were asked to switch off their phone, and to try to focus on the task as much as they could. If participants normally wore glasses for close vision they were asked to wear them. Participants also were asked to avoid overthinking or rushing, and to complete the tasks at their normal pace. Participants were further advised that they could withdraw from the study without penalty at any point if they so wished.

The procedure was approved by the local ethics committee, and all aspects of the study conformed to the Helsinki declaration.

### Results

#### WCST

Table 1 shows descriptive statistics for the two groupsof participants on the WCST. We first ran non-parametric tests on each of the performance measures (Total Errors, Perseverative Errors, Set Loss Errors) to observe whether and how they were affected by age. We also performed correlational analyses between the error types to explore potential associations between them. We then analyzed mean response times for trials following positive and negative feedback, by performing a log-transformed 2 × 2 ANOVA, with age group as a between-subjects factor and feedback as a within-subjects factor. This was primarily to examine the interaction between age group and feedback type for response time.

**Table 1.** Means (and standard deviations) for key dependent measures on the WCST for the two groups of participants

	CA	PE	$SL_{3}$	TE
*Younger*	6.2 (SD = 1.9)	11.3 (SD = 4.29)	0.64 (SD = 1.00)	17.0 (SD = 5.59)
*Older*	4.5 (SD = 2.8)	13.3 (SD = 6.40)	1.48 (SD = 1.56)	20.9 (SD = 8.97)

##### Performance measures

All dependent measures were non-normally distribution, and significantly so (Shapiro–Walk test: p<.001 in all cases). Given the relatively small sample, the non-normality, and the poor response to data transformation, we proceeded with non-parametric tests for the performance variables. In addition to the traditional statistics, we calculated the Bayes Factor ($BF_{10}$) using a Cauchy distribution centered on zero and with scale $\gamma =\frac{1}{\sqrt{2}}$ as a prior. The value indicates how many times the alternative hypothesis is more likely than the point null hypothesis (Wagenmakers et al., 2018). Independent two-tailed Mann–Whitney tests were conducted between groups for each dependent variable.

Comparing Total Errors in Younger (M=17, SD=5.59) and Older (M=20.9, SD=8.97) participants revealed that the difference was not significant (W=250.5, p=.231, $BF_{10}=0.51$). Comparing Perseverative Errors in Younger (M=11.3, SD=4.29) and Older (M=13.3, SD=6.40) participants also revealed that this difference was not significant (W=291.5, p=.688, $BF_{10}=0.33$). However, Set Loss errors were found to be significantly less frequent in Younger (M=0.64, SD=1.00) participants than in Older (M=1.48, SD=1.56) participants (W=202.0, p=.023), albeit with a $BF_{10}$ only marginally higher than 1, $BF_{10}=1.34$ (*robust*). Similarly (and perhaps as a consequence), the number of Categories Achieved by Younger (M=6.2, SD=1.9) participants was significantly greater than that of Older (M=4.5, SD=2.8) participants (W=411.5, p=.049, $BF_{10}=1.34$).

Consistent with the results of these between-subjects comparisons, the correlation between age in years and PE was not significant (r=.167, p=.247, *two-tailed*), but the correlation between Age and $SL_{3}$ was (r=.349, p=.013, *two-tailed*). There was also a significant correlation between PE and $SL_{3}$ (r=.462, p<.001, *two-tailed*), and this correlation remained significant when the effects of Age were partialled out (r=.406, p=.004, *two-tailed*).

##### Response times

**Figure 1.**
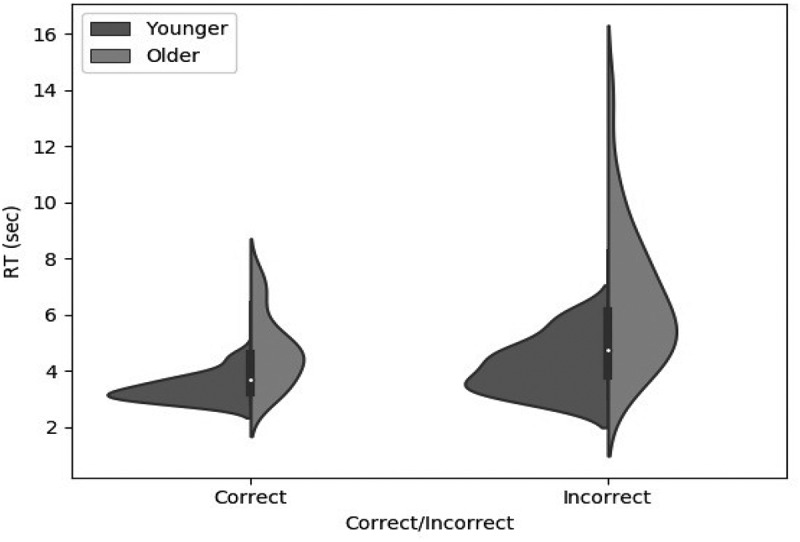
Response time violin plot as a function of age group in the WCST, on trials following “correct” versus “incorrect” feedback. Older participants were in general slower than younger participants, and both groups were slower following negative than positive feedback.

Figure 1 shows mean response time as a function of age and feedback on the previous trial. A two-way ANOVA on the log-transformed data showed that Older participants took significantly longer than Younger participants ($F\left(1,48\right)=31.43$, p<.001, $\eta_{p}^{2}=.396$), and that response time was significantly longer on the trial after an incorrect response than on the trial after a correct response ($F\left(1,48\right)=65.13$, p<.001, $\eta_{p}^{2}=.150$). The interaction between age group and feedback approached significance ($F\left(1,48\right)=3.34$, p=.074, $\eta_{p}^{2}=.008$), but Bayesian analysis showed that including the interaction term did not bring any substantive improvement to the model ($\Delta BF_{10}=0.076$).

#### BRXT

Possibly due to a misunderstanding of the instructions, one older participant did not give any correct answer in the BRXT, and was therefore excluded from the analysis of the BRXT data. The data reported here is therefore from 25 younger participants and 24 older participants. Table 2 shows descriptive statistics for the two groups of participants on the BRXT. We ran non-parametric tests on each of the performance measures (total errors, perseverative response errors, perseverative stimulus errors, perseverative rule errors) to observe whether and how age affected them. We also performed correlational analyses between the error types to explore potential associations between them. Like the WCST, we then analyzed response times by performing a log-transformed 2×2 ANOVA, with age group as a between-subjects factor and feedback type as a within-subjects factor, again primarily to examine the interaction between age group and feedback type on response times.

**Table 2.** Means (and standard deviations) for key dependent measures on the BRXT for each group of participants

	$P_{RESP}$	$P_{STIM}$	$P_{RULE}$	TE
*Younger*	1.36 (SD = 1.04)	0.44 (SD = 0.91)	4.16 (SD = 1.48)	10.44 (SD = 4.74)
*Older*	1.63 (SD = 1.74)	0.17 (SD = 0.64)	3.79 (SD = 0.78)	13.83 (SD = 7.03)

##### Performance measures

All performance variables analyzed (total errors, perseverative response errors, perseverative stimulus errors, perseverative rules errors) were not normally distributed, as shown by a Shapiro–Wilk test (p<.001). Again, given the relatively small sample, the non-normality, and the poor response to data transformation, we used non-parametric tests to analyze these performance variables. In addition to the traditional statistics, we again calculated the Bayes Factor ($BF_{10}$) using a Cauchy distribution centered on 0 and with scale $\gamma =\frac{1}{\sqrt{2}}$ as a prior.

Independent two-tailed Mann–Whitney tests were conducted. The difference in total errors produced by Younger participants (M=10.44, SD=4.74) and Older participants (M=13.83, SD=7.03) was significant (W=202.1, p=.024, $BF_{10}=1.53$ (*robust*)). However, the difference in perseverative response errors in Younger (M=1.36, SD=1.04) and Older (M=1.63, *SD *= 1.74) participants was not significant (W=298.0, p=.487, $BF_{10}=0.31$). Similarly, the number of perseverative stimulus errors produced by Younger (M=0.44, SD=0.9) and Older (M=0.17, SD=0.64) participants did not differ (W=357.5, p=.957, $BF_{01}=0.49$), and neither did the number of perseverative rule errors (Younger: M=4.16, SD=1.48; Older: M=3.79, SD=0.78; W=355.0, p=.895, $BF_{10}=0.45$).

Correlational analysis revealed that while total errors correlated significantly with Age in years (r=.297, p=.038), this was not reflected in any of the more specific perseverative error measures (Age against $P_{RESP}$: r=.091, p=.535; Age against $P_{STIM}$: r=−.119, p=.415; Age against $P_{RULE}$: r=−.157, p=.283). However, across all participants the correlation between $P_{RESP}$ and $P_{STIM}$ was significant (r=.345, p=.015), while the correlations between $P_{RULE}$ and $P_{RESP}$ (r=−.007, p=.964) and $P_{RULE}$ and $P_{STIM}$ (r=−.109, p=.456) were not.

##### Response times

**Figure 2.**
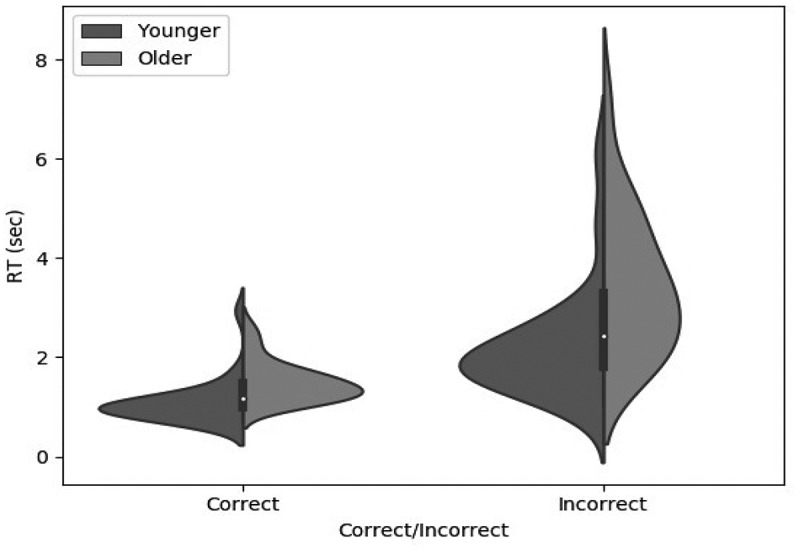
Response time violin plot as a function of age group in the BRXT, on trials following “correct” versus “incorrect” feedback. Older participants were in general slower than younger participants, and both groups were slower following negative than positive feedback.

Figure 2 shows the mean response time across trials as a function of age group and feedback on the previous trial. A two-way ANOVA on the log-transformed data showed that Older participants took significantly longer than Younger participants ($F\left(1,47\right)=22.64$, p<.001, $\eta_{p}^{2}=.325$), and that response time was significantly longer after an incorrect response than after a correct response ($F\left(1,47\right)=216.89$, p<.001, $\eta_{p}^{2}=.822$). The interaction between the effects of age and feedback was not significant ($F\left(1,48\right)=1.12$, p=.294), and Bayesian analysis showed that including the interaction term did not bring any substantive improvement to the model ($\Delta BF_{10}=-0.055$).

#### Cross-task correlations

The correlation (across all participants) between the total number of errors on the WCST and on the BRXT was significant and positive (r=.359, p=.011, two−tailed). This correlation remained significant when the effect of age was partialled out (r=.308, p=.033, two−tailed).

Table 3 shows the correlations between the numbers of each type of error (across all participants) for each task. While none of the correlations between SL_3_ on the WCST and any of the BRXT perseverative error types are significant, PE on the WCST correlates significantly with P_RESP_ and P_STIM_ on the BRXT, but not with P_RULE_ Given the small sample size and the relevance to the discussion, we computed the Bayes Factor for three of these correlations. Bayesian analysis using the aforementioned priors yielded a *BF*_10_ of 5.94, 3.32, and a *BF*_01_ of 1.53 for PE correlations with *P_RESP_, P_STIM_*, and *P_RULE_*, respectively.

**Table 3.** Pearson correlations between types of WCST error (rows) and BRXT error (columns). All probabilities are two-tailed

	P_RESP_	P_STIM_	P_RULE_	TE
PE	*r* = .367	*r* = .322	*r* = −.088	*r* = .384
	*p* = .009*	*p* = .024*	*p* = .547	*p* = .006*
SL_3_	*r* = .104	*r* = −.006	*r* = −.110	*r* = .535
	*p* = .477	*p* = .968	*p* = .454	*p* = .001*

*Note*. Values of p < .05 are marked with *

### Discussion

In the WCST, we found that older participants produced more set loss errors, but not more perseverative errors, than younger participants. The significant increase in set loss errors with age is consistent with the detrimental effect of aging in proactive control, conceptualized as the process by which information in working memory biases attention toward goal-relevant schemas (Braver, 2012). Thus, we attribute the tendency toward increased set loss errors in older participants to a reduction with age in the ability to maintain or sustain attention to an ongoing task. This interpretation for the origin of set loss errors is consistent with, for example, that of Stuss et al. (2000) and deriving from the neuropsychological literature, who found that patients with inferior medial frontal lesions (as opposed to those with superior medial frontal lesions or with lateral lesions) were particularly prone to producing such errors. They attributed this to a deficit in sustained attention to an ongoing task (see also Shallice, Stuss, Picton, Alexander, & Gillingham, 2008; Stuss et al., 2005).

The absence of a significant effect of age on perserverative errors within the WCST was also predicted, though only on the assumption that our older participants were unrepresentative of the general population. Older participants recruited through age charities, and in particular the University of the Third Age (which characterizes itself as “an international movement whose aims are the education and stimulation of mainly retired members of the community”), are unlikely to constitute a representative sample of the elderly population in that such participants are likely to be more active and better educated than average. This interpretation is consistent with prior research cited above, but is of particular interest when considered in conjunction with the age effect on set loss errors, for together the results show that, at least in this population, the two error types dissociate.

Results from the BRXT were also consistent both with prior literature (e.g., Bielak et al., 2006; Esther et al., 2009) and the above interpretation of the WCST results, in that there was no statistical difference between the number of perseverative errors made by older participants and younger participants, while the total number of errors made by older participants was significantly greater than that made by younger participants. Recall that prior literature concerned with this task and aging has focussed only on total errors. Thus, Bielak et al. (2006) reported that while their 50–59 year olds (N=55) produced 16.5 errors on average (SD=6.7), their 70–79 year olds (N = 97) produced 20.5 errors on average (SD=7.0). Similarly, Esther et al. (2009), who also assessed years of education, derived the following regression equation relating age to total errors:

$$\hat{TE}=6.12+\left(0.23\times Age\right)-\left(0.24\times Years\;of\;Education\right)$$

This was based on a group of 283 healthy controls with mean age 67.4. On this equation, a 65 year old adult with 14 years education would be predicted to produce 17.71 errors, while an education-matched 85 year old would be predicted to produce 22.31 errors. While the absolute total error scores in these two studies are higher than observed in the current study, their relative values are consistent with the pattern observed here. Absolute differences may be explained by procedural differences in the current study (e.g., our use of a circular arrangement of disks in place of the 5×2 rectangular array, and the use of 9, rather than 10, disks). The absence of any significant effect of age on perseverative errors in the current study suggests that it is processes related to the production of non-perseverative errors, such as those involved in sustaining attention to the current goal, maintaining representations of recent trials, and inducing rules via the identification of temporospatial patterns across those representations, that are affected by aging.

With regard to response times, as expected, in both tasks latency to response was greater on trials following incorrect responses (and hence negative feedback) than on trials following correct responses (and hence positive feedback), and for older participants than for younger participants. However, while the difference between response time after correct and incorrect responses was numerically larger in both tasks for older participants than younger participants, the interaction effect was not significant. The slowed response times following negative feedback presumably reflect processes related to response inhibition and subsequent rule induction. These processes would seem to be shared by the two tasks.

Turning to the relation between performance on the WCST and the BRXT, the main outcomes of our correlational analyses were that (across participants of all ages): a) PE in WCST is predicted by $P_{RESP}$ (and to a lesser extent $P_{STIM}$) in BRXT (or vice versa), but not by $P_{RULE}$; and b) $SL_{3}$ in WCST is predicted by TE in BRXT (or vice versa), but not by any of the three measures of BRXT perseverative errors. The former suggests that the processes underlying production of perseverative errors in the WCST are related to those underlying production of stimulus or response perseveration in the BRXT (which were also found to correlate), but not perseveration of rule application, while the latter further supports the claim that set loss errors and perseverative errors have different origins, or more precisely, that set loss errors in the WCST are the result of (failure of) processes that are separable from those involved in the production (or avoidance) of perseverative errors.

## Computational modeling

In the second part of this paper, we consider how a recently developed neurocomputational model of schema-driven behavior, that of Caso & Cooper (2020), which has previously been used to help understand the deficit of Parkinson’s patients on the WCST, can be applied to our data to elucidate how putative neurobiological effects of aging might impact upon the learning and performance of rule-based tasks. The model has been described in full elsewhere. Therefore, rather than repeat that description here, we instead focus only on the aspects of the model critical to understanding its application to the two tasks considered here.^1^

### Model description

The model is based on the work of Norman & Shallice (1986), who developed a schema-theoretic account of sequential action selection. Their theory proposes that hierarchically organized action schemas – abstractions over instances of action sequences – compete with each other through activation-based processes for control of action. This functional description is not committed to a specific neural implementation or a specific task, but application of the model to a specific task (e.g., the WCST or the BRXT) may be achieved by providing the model with appropriate task-specific schemas.

#### Model structure and operation

**Figure 3.**
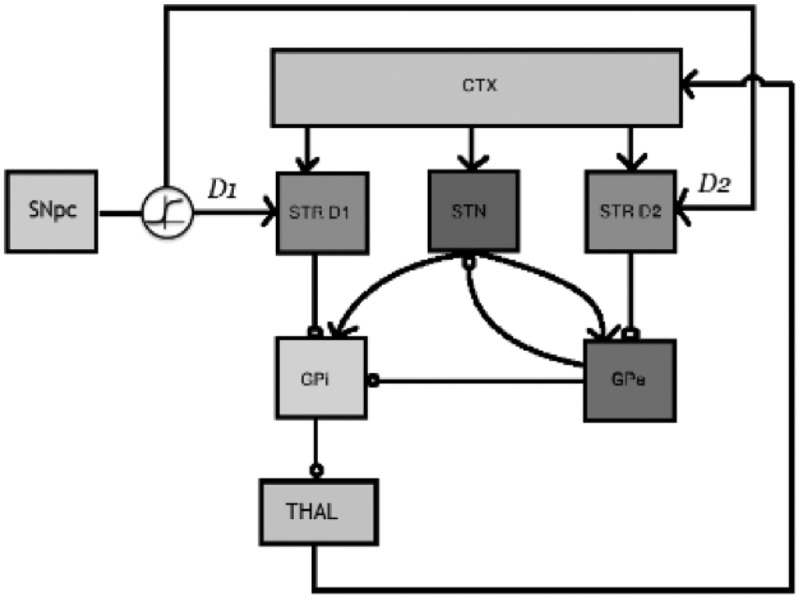
Schematic of the basal ganglia. STR D1: Striatum D1 receptors, STN: Subthalamic Nucleus, STR D2: Striatum D2 receptors, GPi: Globus pallidus internal segment, GPe: Globus Pallidus external segment, THAL: Thalamus, CTX: Cortex, SNpc: Substantia Nigra pars compacta. Standard arrow heads indicate excitatory connections. Circular arrow heads indicate inhibitory connections.

Our general model (see Caso & Cooper, 2020, for full details) features two sets of schemas: cognitive schemas and motor schemas (conceptualized as localized in prefrontal and premotor areas, respectively). The former encode higher level regularities of a task (e.g., sort by color, or alternate between positions) while the latter correspond to specific sensorimotor or response schemas (e.g., select pile 1 in the WCST, or select the disk at the “4 o’clock” location in the BRXT). Each schema has an associated activation value that varies between zero and one and that is maintained by a simulated cortico-thalamic loop. Each such loop comprises a schema node and a set of nodes corresponding to the nuclei of the basal ganglia, following the functional organization of the neuroanatomically detailed model of Gurney, Prescott, & Redgrave (2001), as shown in Figure 3. The basal ganglia units implement the multiple sequential probability ratio test (Bogacz & Gurney, 2007), an optimal method of simultaneously testing multiple hypotheses by estimating the probability of each on the basis of their support as judged by the input data.

All units function according to standard activation-based processing principles, with the activation of each unit at any point in time being a function of the weighted sum of its inputs, and with the saturation function by which a node’s activation is calculated being the standard logistic function:

(1) $$\begin{array}{c} \sigma_{\beta ,\alpha }\left(x\right)=\frac{1}{1+e^{-\alpha \cdot \left(x-\beta \right)}} \end{array}$$

where x is the unit’s summed weighted input, and α and β are parameters that control the unit’s gain and threshold, respectively.

Each cortico-thalamic loop operates in parallel, with the organization of Figure 3 ensuring tonic inhibition of all schema nodes through the inhibitory connection from each schema’s globus pallidus internal segment (GPi) unit to its thalamus (THAL) unit. In addition, however, each schema’s GPi unit also receives excitation from the output of the subthalamic nuclei (STN) units of *all* schemas within each competing set. Because unit activation is thresholded (as per Equation 1), this acts to release tonic inhibition from the more active schema nodes, allowing the activation of those nodes to increase. With appropriate parameter settings the dynamics of the system ensures that at any point in time only one schema node from each set is highly active. If this activation is sustained, the schema is then selected, and begins to control behavior. If, subsequently, the threshold of that node is increased or its gain is decreased (e.g., as a result of negative feedback), the node’s activation will reduce, allowing some other node within the set to become active.

As described thus far, schemas within each set compete with each other. Thus, cognitive-level schemas compete with each other and sensorimotor schemas compete with each other. Schemas across sets, however, cooperate. Thus, cognitive-level schema nodes may also excite corresponding sensorimotor schema nodes, with that excitation dependent on schema selection at the cognitive level and the input stimulus (e.g., if in the WCST “sort by color” is selected and the color of stimulus card is red, then excitation will be passed from the “sort by color” node to the sensorimotor schema node corresponding to the red target location).

#### Cortical learning

We assume that the slope or gain of the saturation functions of cortical units dynamically adapts to the level of conflict between those units. In particular, when the activation of several cortical representations is very similar, and the basal ganglia alone cannot arbitrate between different representations (e.g., because feedback/reward has not yet been received and computed), a mechanism is required to resolve this conflict so as to allow the simulated basal ganglia to make a decision. At the same time, the stability of cognitive representations must be sensitive to the need to trade off exploration and exploitation at different levels of the schema hierarchy (Goschke & Bolte, 2014). Allowing the gain of the saturation functions at each level of the schema hierarchy to vary in response to conflict provides a mechanism for this.

Here, we implement a mechanism that allows the cortical sensorimotor/response units to change the gain/slope of their saturation function, $\alpha_{sma}$, via the free parameter *ε_sms_* according to Equation 2:

(2) $$\alpha_{sma}\leftarrow \left(1+\zeta_{sma}\right)\overset{N}{\underset{i}{\prod }}\left(1+\varepsilon_{sma}+o_{sma,i}\right)$$

where $\zeta_{sma}$ is sensorimotor unit noise, the product is over all sensorimotor units, and $o_{sma,i}$ is the output signal of the $i^{th}$ sensorimotor unit. For simplicity, we do not include the analogous dynamic slope adjustment for cognitive schemas.

Increasing the $\alpha_{sma}$ parameter is comparable to being more confident at responding, provided that the subject has accrued the same level of evidence and has the properties desired of a conflict construct, as shown by Berlyne (1957; see also Botvinick, Braver, Barch, Carter, & Cohen, 2001). Conflict should increase when the number and the activation of competing representations increase, and it should have its maximum value once all units reach theirs. Many functions satisfy these criteria, but the product of activation values is the simplest analytical form, and it is therefore the one we use here. The presence of conflict stabilizes or destabilizes schema activation as a function of their input, driving a change to schema activation values in a dynamic fashion. The change of slope of the saturation function in the sensorimotor units provides this implementation. Cognitive control can be construed as a detection mechanism of suboptimal performance (or anticipation of such performance in the case of proactive control), followed by a change in attentional focus. At the level of evidence accumulation, conflict resolution can be achieved by slowing the accumulation of activation in specific units. The value of $\alpha_{sma}$ is updated each time feedback is received.

#### Basal ganglia learning

The basal ganglia units are regulated in a different fashion from the cortical units. While cortical units are solely regulated by their online state, regardless of history of activation and external stimuli, basal ganglia units change their characteristics with a history-based and reward-driven time course. Within the model this is reflected by adjusting $\beta_{str}$, the threshold of the saturation function in striatal units, which is assumed to be related to the level of striatal dopamine. On each processing cycle and within each cortico-thalamic loop, i, $\beta_{str,i}$ is adjusted according to Equation 3:

(3) $$\beta_{str,i}\leftarrow \left(\beta_{str,i}-\varepsilon_{str}\cdot \delta_{i}\right)\cdot \left(1+\zeta_{str,i}\right)$$

where the calculated value of $\beta_{str,i}$ is clipped to within the range [0, 1] if it falls below 0 or above 1.

The $\delta_{i}$ in Equation 3 is the reward prediction error, expressed as the difference between the actual reward $f_{i}$ and the median activation value in the last trial $a_{i}$, as per Equation 4:

(4) $$\delta_{i}\leftarrow r\cdot \left(f_{i}-a_{i}\right)$$

where r is either +1 or −1, according to whether feedback following the previous action/response was positive (e.g., a correct answer) or negative (e.g., an incorrect answer).

In Equation 4, $f_{i}$ is calculated as follows:

(5) $$f_{i}\leftarrow \left\{\begin{array}{cc} +1 & matching\;schemas\\ \left(2w_{neg}-1\right)-\left(m_{r}\cdot f_{i}^{t-1}\cdot r^{t-1}\right) & mismatching\;schemas \end{array}\right.$$

The t−1 index indicates the values of the variables on the previous iteration, while the parameters $w_{neg}$ and $m_{r}$ determine the reward for mismatching schemas. If $w_{neg}$ (*mismatch reward sensitivity*) is zero then the baseline reward is −1, meaning that prediction error ($\delta_{i}$ in Equation 3) will be zero when the median activation of schema i on the previous trial is −1. As $w_{neg}$ increases, this baseline increases. The $m_{r}$ parameter (*memory for negative feedback*) determines the extent to which feedback from previous trials modulates the baseline given by $w_{neg}$. If it is zero, feedback from previous trials is ignored, but if it is greater than zero then feedback from previous trials persists across trials.

Together, Equation 3, Equation 4 and Equation 5 allow the model to bias $\beta_{str}$ in the correct direction following feedback.

#### Key model parameters

The model has a relatively large number of parameters (see Caso & Cooper, 2020, for a complete list), but in the simulations reported here we consider four of them as free parameters: mismatch reward sensitivity ($w_{neg}$), memory for negative feedback ($m_{r}$), striatal learning rate (*ε_str_*) and sensorimotor schema learning rate (*ε_sma_*). As noted above, the first two parameters affect the sensitivity of mismatching schemas to feedback. When $w_{neg}$ and $m_{r}$ are zero, $f_{i}$ in is −1 for mis-matching schemas, meaning that if the correct schema was selected (so r is +1), the thresholds of mismatching schemas will be increased (making them even less likely to be selected), while if the incorrect schema is selected (so r is −1), the thresholds of mis-matching schemas will be decreased, making them more likely to be selected. As mismatch reward sensitivity ($w_{neg}$) increases, the magnitude of the threshold adjustment for mismatching schemas will decrease. Moreover, as memory for negative feedback ($m_{r}$) increases, the threshold adjustment for mismatching schemas is increasingly modulated by reward on the previous trial. The third and fourth parameters affect the efficacy of learning in the model’s striatal and the cortical components. The striatal learning rate (*ε_str_*) controls the rate of learning of biases/thresholds in the activation functions of the basal ganglia units for the cognitive schemas, while the sensorimotor schema learning rate (*ε_sma_*) modulates the extent to which the information entropy of sensorimotor schemas drives the change of slope in their activation functions.

### Relation to other neurobiological models

Rule induction tasks such as the WCST, and frontal functioning more generally, have been the target of a number of computational models, both at the cognitive level and the neurobiological level. Our model bears similarities to some of these existing models, but also incorporates notable differences. Bishara et al. (2010)’s cognitive-level of the WCST, for example, effectively maintains a representation of the probability of each rule (or more specifically each stimulus dimension) controlling the response, with this representation being updated following feedback such that the probabilities of rules consistent with the response/feedback are increased and the probabilities of rules inconsistent with the response/feedback are decreased following each trial. Model parameters determine sensitivity to reward, punishment, and decision consistency. The model has subsequently been applied to clinical data from a range of neurological conditions (e.g., Parkinsonism: Steinke, Lange, Seer, & Kopp, 2018), with moderate success. Response/feedback pairs in our model can similarly be understood as providing evidence for or against rules, but unlike the Bishara et al. (2010) model, our model operates at a more mechanistic level (by incorporating a neurobiologically plausible model of the basal ganglia) and incorporates lower-level sensorimotor schemas. Such low-level schemas effectively provide our model with a direct route whereby a stimulus can drive a response in the absence of top-down input.

An alternative cognitive-level account of rule induction is provided by Steinke, Lange, & Kopp (2020), who provide a reinforcement learning model of the WCST which combines model-free (habitual) and model-based (deliberative) subsystems. Like the model of Bishara et al. (2010), Steinke et al. (2020)’s model produces a probability distribution over responses on each trial, and like the model of Bishara et al. (2010) it produces perseverative errors because negative feedback does not reduce the probability of applying the previously most likely rule to zero, while it produces set loss errors because even with positive feedback there is a non-zero probability of failing to apply the most probable rule. The innovation of the Steinke et al. (2020) model lies in its combination of model-based and model-free “routes” to action. This is based on the authors’ demonstration of learning at the response level, whereby detailed analysis of behavioral data suggests that negative feedback results in an increased likelihood of participants avoiding the specific (punished) response on the next trial. Steinke et al. (2020) argue that their model provides a superior account, compared with the model of Bishara et al. (2010). The reinforcement learning model does not directly refer to the operation of cortico-subcortical loops, but nevertheless obtains a neurobiological interpretation through the identification of model-based processing with frontal functions and model-free processing with habitual action selection primarily associated with the basal ganglia (e.g., Daw, Niv, & Dayan, 2006). The model therefore bears a more abstract relation to neural processes than our model presented here, though both models include elements of learning from reinforcement (in our model, through Equation 4.) More crucially both models include two levels of processing – model-based processing is equivalent to application of rule schemas while model-free processing is equivalent to application of sensorimotor schemas. It is, however, beyond the scope of the current work to determine whether or how our model might capture the specific effects of perseverative response avoidance (what they term “the modulation of perseveration propensity by response demands”) seen by Steinke et al. (2020) to be a critical aspect of behavior.

Several other authors have also developed neurobiologically-grounded models of rule induction tasks which incorporate cortico-subcortical loops (e.g., Frank & Badre, 2012; Monchi, Taylor, & Dagher, 2000), and the operation of striatal learning (Equation 3, Equation 4, and Equation 5) in our model bears a resemblance to those present in that proposed by Frank & Badre (2012). However, our model differs from that of Frank & Badre (2012) in several substantial details. First, our model assumes that participants come to tasks such as the WCST with schemas for the classification of objects based on visually distinct dimensions (such as number, color, and form). This is similar to the assumption of Monchi et al. (2000), but connectionist network models such as that of Rougier & O’Reilly (2002) (and subsequently that of Frank & Badre, 2012) achieve this by pretraining the model with color, form, and number classification subtasks (see also Rougier, Noelle, Braver, Cohen, & O’Reilly, 2005). Perhaps more critically, in our model, the basal ganglia do not gate information through a series of weights adjusted by experience (in order words, it is not a connectionist network in the PDP tradition). Rather, the basal ganglia implement a control system that operates on time-continuous variables. Furthermore, while motor and premotor areas are separated and hierarchically related (i.e., information is passed downward) in both our model and that of Frank & Badre (2012), our model does not distinguish between a maintenance layer, deep lamina layer, and even a visual input layer, but instead incorporates all of them in sensorimotor schemas.

Most recently, Barceló (2020) has argued for a reframing of the WCST in terms of the predictive coding framework of Friston (2009). This approach argues that the function of the brain is to “infer the causes of its sensory inputs.” When applied to the WCST and rule induction more generally, the predictive processing approach attempts to infer the underlying rule by minimizing unanticipated feedback. Barceló (2020) argues that this results in two phases of operation. Early in the induction process, when there is greatest uncertainty in the rule, the system must explore the various options, while later in the induction process, when the rule is known, the system must exploit its knowledge (and predictions). These two phases are held by Barceló (2020) to be reflected in electrophysiological studies which have found different ERP profiles associated with the early and late trials within each run of a rule, with early processing being associated with prefrontal negativity and later processing associated with more posterior (and possibly parietal or subcortical) negativity (e.g., Lange, Seer, & Kopp, 2017; Stuss & Picton, 1978). The explore/exploit dimension has echoes of reinforcement learning, but within the predictive processing account exploration is not unguided or random, but aimed at reducing the uncertainty in the current rule, thereby improving the generative model guiding behavior, and decreasing the likelihood of negative feedback. An assumption that is common to our approach and the predictive processing approach is that both effectively rely on predicting feedback and attempting to minimize prediction error – note that on the assumption that activation in our model encodes an expectation, the central term in Equation 4, $f_{i}-a_{i}$, represents the error between actual reward feedback and anticipated feedback. Hence, when prediction error is zero, changes in striatal gain occur only as a result of noise. Indeed, ongoing changes in striatal gain during the latter stages of a run of trials with the same rule, through Equation 4, work to reduce prediction error.

### Simulation 1: modeling the WCST

#### Introduction: application of the model to the WCST

To model performance on the WCST, the model was configured with three cognitive schemas, corresponding to the three WCST rules (sort by color, sort by number, sort by form), and four sensorimotor/response schemas, corresponding to the four target locations where stimulus cards might be placed. When a cognitive schema is selected, activation is passed (via the corresponding response-level STN unit) into the cortico-thalamic loop for the appropriate target location (which is determined with reference to the stimulus card and the selected cognitive schema). If the selection is correct (i.e., experimenter feedback is positive, and so r in Equation 4 is +1), feedback to cognitive-level schemas (as per Equation 5) is positive if at least one feature of the stimulus card matches the selected target card and negative otherwise. Alternatively, if the selection is incorrect (i.e., experimenter feedback is negative, and so r in Equation 4 is −1), feedback to cognitive-level schemas (as per Equation 5) is negative when at least one feature of the stimulus card matches the selected target card and positive otherwise.

#### Method

In order to fit the model to the data from the WCST and to explore the effects of the model parameters, we conducted a series of data-fitting studies, where TE, PE, and $SL_{3}$ were the target variables. More specifically, we sought to minimize the norm of the z scores for the three variables (i.e., the square root of the sum of squared errors for the three variables) by using simulated annealing (Peter JM & Aarts, 1987). Additionally, we calculated the Bayes Factor, $BF_{01}$, for each dependent measure,^2^ given a standard Cauchy prior with $x_{0}=0$ and $\gamma =\frac{1}{\sqrt{2}}$. While there is a negative correlation between z values and $BF_{01}$, the simulated annealing algorithm does not minimize $BF_{01}$ efficiently, but operates better on a function of the z values, perhaps owing to the fact that the BF space is less smooth than the z space, and it is therefore easier when minimizing $BF_{01}$ to get stuck in local minima. Therefore, if two parameter sets yielded a similarly low z value, the one with higher BF was chosen. This additional criterion improves model selection by taking into account distributions as well as providing a more intuitive fitting index.

We found the best-fitting values for each age group separately. In each case, simulated annealing was initialized with a parameter set obtained through qualitative analysis to ensure faster convergence. Moreover, we observed that for both groups very good fits were obtained with $m_{r}$ at its default value of zero, and so this parameter was fixed at this value while the other three parameters were varied in order to fit the group data.

#### Results

**Figure 4.**
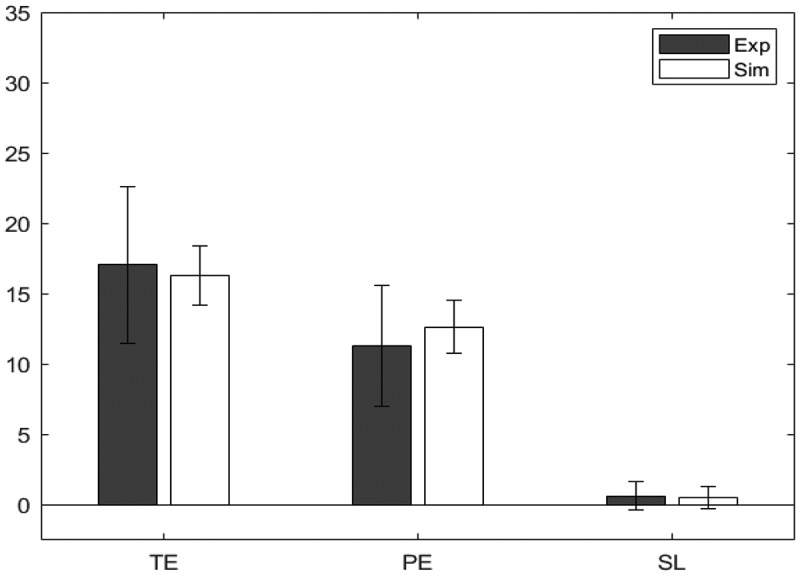
Model fit for younger participants, comparing the experimental (Exp) values and the simulated (Sim) values for the WCST.

**Figure 5.**
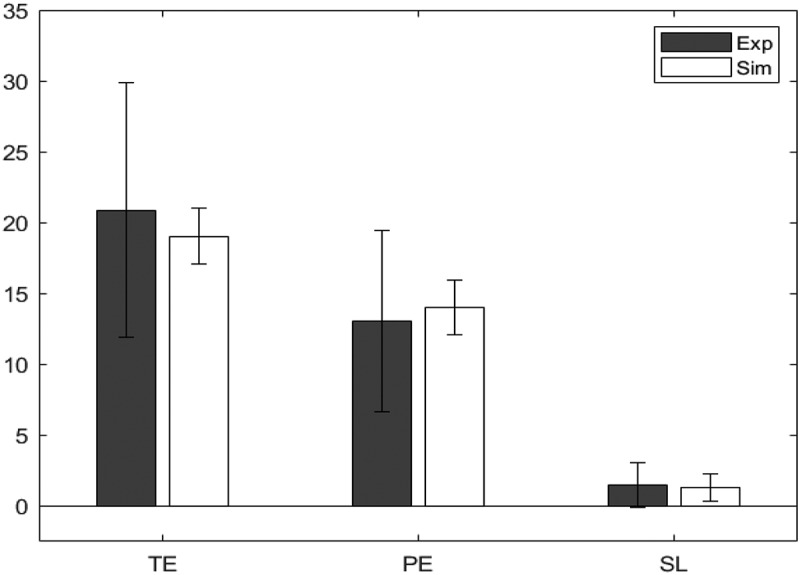
Model fit for older participants, comparing the experimental (Exp) values and the simulated (Sim) values for the WCST.

Table 4 shows the minimized absolute z scores for the three dependent measures, across the two groups. A value closer to 0 indicates a better fit. Also shown in the table are Bayes Factors for each dependent measure. A value greater than 1 indicates support for the null hypothesis, and therefore a better fit (Wagenmakers et al., 2018). The resultant values of the parameters of interest for the two groups are shown in Table 5, while Figure 4 and Figure 5 show bar charts for the dependent measures for the experimental and simulated younger and older participants, respectively.

**Table 4.** Z statistics for the model fit for the two age groups in the WCST. A value closer to 0 indicates that experimental and simulated values have closer means, and therefore a better model fit. The subscripts near the z value are the Bayes Factor (BF_01_). A value higher than 1 indicates greater support for the null hypothesis, which suggests a good model fit

	TE	PE	$SL_{3}$
*Younger*	$0.019_{2.99}$	$0.095_{1.57}$	$0.015_{3.22}$
*Older*	$0.042_{2.35}$	$0.021_{2.91}$	$0.011_{3.27}$

**Table 5.** Values of the parameters $w_{neg}$, $m_{r}$, ε_str_, and ε_sma_ for the simulations of the two age groups in the WCST

	$w_{neg}$	$m_{r}$	*ε_str_*	*ε_sma_*
*Younger*	.457	.000	.139	.833
*Older*	.106	.000	.097	.000

Importantly, swapping the parameter values determined for each age group results in substantially poorer overall fits for both groups, with larger z values, and $BF_{01}$ values generally smaller than 1 (see Table 6). This suggests that the model adequately simulates the responses of the two participant groups as being distinct.

Z (and Bayes Factor $BF_{01}$ as a subscript) for model fits obtained by swapping young and old simulated data. The overall increase in Z values and the presence of four BFs below one suggest that the two models are not interchangeable and they capture the differences between the two groups.

**Table 6.** Z and Bayes Factor BF_01_ as a subscript for model fits obtained by swapping young and old simulated data. The overall increase in Z values and the presence of three BF_s_ below zeros suggest that the two models are not interchangeable and they capture the differences between the two groups

	TE	PE	SL_3_
$Y_{exp}$, $O_{sim}$	$.128_{1.12}$	$.391_{0.15}$	$467_{0.34}$
$O_{exp}$, $Y_{sim}$	.263_0.30_	$.005_{3.38}$	$.380_{0.18}$

Recall that in the experimental study we found that response time was affected by both age and feedback on the previous trial, with older participants slower than younger ones, and responses after negative feedback slower than responses after positive feedback. Table 7 shows the number of cycles to selection of a sorting action for the model given the parameter settings determined for younger and older groups, and as a function of feedback on the previous trial. The simulations reproduce the effect of age ($F\left(1,48\right)=144.062$, p<0.001), with the simulated older participant group taking more cycles to respond than the simulated younger participant group, but not the effect of feedback type ($F\left(1,48\right)=1.435$, p=0.237). Moreover, the factors do not interact ($F\left(1,48\right)=1.257$, p=0.268).

**Table 7.** Mean (standard deviation) simulated response time (i.e., number of cycles to generate a response) for each simulated group for trials following positive feedback and trials following negative feedback. Data are based on 25 simulated runs with parameters set as in Table 5

	Positive	Negative
*Younger*	132.3 (1.4)	132.2 (2.9)
*Older*	146.9 (5.5)	144.5 (8.8)

#### Discussion

The model is able to fit, near perfectly, the error data of both participant groups. Despite a similar number of perseverative errors in both groups, older participants are simulated with a reduction of 0.042 in the value of the striatal learning rate (*ε_str_*, down from 0.139 to 0.097), which suggests a slower update of basal ganglia thresholds in older participants than in younger participants. The corresponding reduction from 0.833 to 0.000 in the sensorimotor learning rate (*ε_sma_*) reflects instead a reduction in cognitive control. With regard to mismatch reward sensitivity, the value of $w_{neg}$ for younger participants (of 0.457) suggests substantial insensitivity to negative feedback. The value for older participants (of 0.106) suggests that older individuals are more sensitive to negative feedback than their younger counterparts. Since the fit was obtained with $m_{r}$ fixed at zero for both groups, these results assume that both groups are insensitive to reward from previous trials – an assumption that appears reasonable given the fits obtained.

With respect to the response time data, the model accounts well for the difference in response time due to age, but not for the difference due to feedback type. This contrasts with our earlier application of the model to data from Parkinson’s patients on a slightly modified version of the WCST (Caso & Cooper, 2020), where the number of model cycles to a response was greater following negative feedback than following positive feedback. The lack of effect in the model behavior here is in part due to a difference in the version of the WCST and differences in parameter values. More specifically, the version considered in the earlier work followed that of Barceló (2003) and included only stimulus cards that matched target cards on a single feature. The version considered here includes all possible cards, including ones which match target cards on two and even three features. The difference in RT on trials following positive and negative feedback is less when ambiguous cards are present, possibly because negative feedback after a card that matches on two dimensions (i.e., ambiguous cards) allows the model to effectively and efficiently rule out both dimensions on which the card matches. The difference in RT also depends in a nontransparent way on the values of the parameters (and in the simulations reported here is positive in some regions of the parameter space and negative in others). We return to this issue below in the discussion of simulation 3.

### Simulation 2: modeling the BRXT

#### Introduction: application of the model to the BRXT

**Figure 6.**
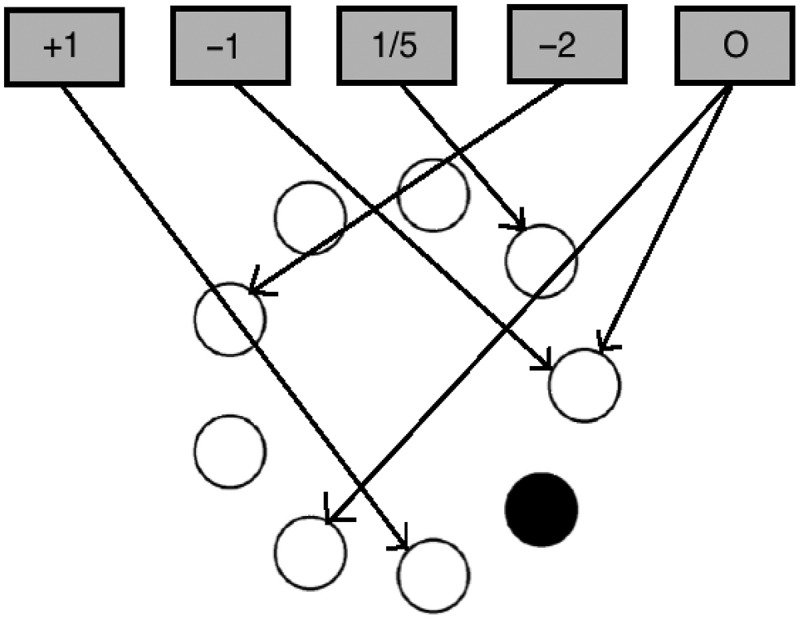
Schematic of the BRXT model without the basal ganglia arbitration device. For instance, given that specific filled-in circle as an input, the +1 schema (clockwise) excites the following circle, whereas the −1 schema (counter-clockwise) excites the preceding one.

To model performance on the BRXT, the model was configured with five high-level schemas and nine lower-level sensorimotor schemas (see Figure 6). Four of the high-level schemas represented the four possible rules from the empirical study (clockwise, counter clockwise, alternate between positions 1–5, counter clockwise skipping one circle). One additional schema was included to represent all the other potential rules. All high-level schemas were fed with a constant input and uniformly distributed noise. The nine lower-level sensorimotor schemas represented the nine possible response options. These low-level schemas were also activated by environmental cues (stimuli) in much the same way as low-level schemas in the WCST model were activated by environmental cues. In this way, in absence of top-down control, environmental cues have the potential to drive the choice of pattern.

The present simulation is not concerned with rule inference per se, since this is assumed to happen higher up in the mental processing hierarchy, but is focused instead on the cognitive control of these rules. Thus, we assume that individuals have acquired similar pattern schemas, such as progressing clockwise around a circle, throughout their life. Nevertheless, some additional comment is required on the rule represented by the fifth cognitive schema. This was randomized to a different rule for each simulated participant, and contributes to inter-subject variability in the simulation. Different participants may have different concepts that are not necessarily triggered by the stimuli presented. For example, some individuals might overcomplicate rules and infer that clockwise motion of the circles is mirrored anticlockwise after a semi-circle is completed or infer bizarre responses that do not reflect any of the most common rules that neurologically healthy individuals seem to employ. Adding this schema also produces meaningful variations in responses.

For the BRXT, schema matching (as required for Equation 5) is determined by comparing the two most recent responses (in order) with the patterns corresponding to each of the known rules. If, for example, two consecutive filled disks appear in counter-clockwise fashion, then the schema corresponding to this pattern will be considered to match (and will receive bottom-up activation). If this arrangement also has features in common with the random schema, then the latter will also be considered to match (and be activated). If feedback is positive then the saturation functions of matching schemas are biased to increase the likelihood of them being selected, as per the first clause in Equation 5. The saturation function of non-matching schemas is biased in the opposite way – to decrease that likelihood (as per the second clause in Equation 5). The reverse applies if feedback is negative, ensuring that on the following trial matching rules are less likely to be selected but mis-matching rules are more likely to be selected.

#### Method

The model fitting studies for the BRXT were conducted in the same way as for the WCST, with one exception, namely that when fitting the data from older participants the value of the memory for negative feedback parameter ($m_{r}$) was also allowed to vary (for reasons given below). This time TE, $P_{STIM}$, $P_{RESP}$, and $P_{RULE}$ were the target variables. Again, fitting was performed using simulated annealing (Peter JM & Aarts, 1987), by minimizing the norm of the z values of the four target variables.

#### Results

**Figure 7.**
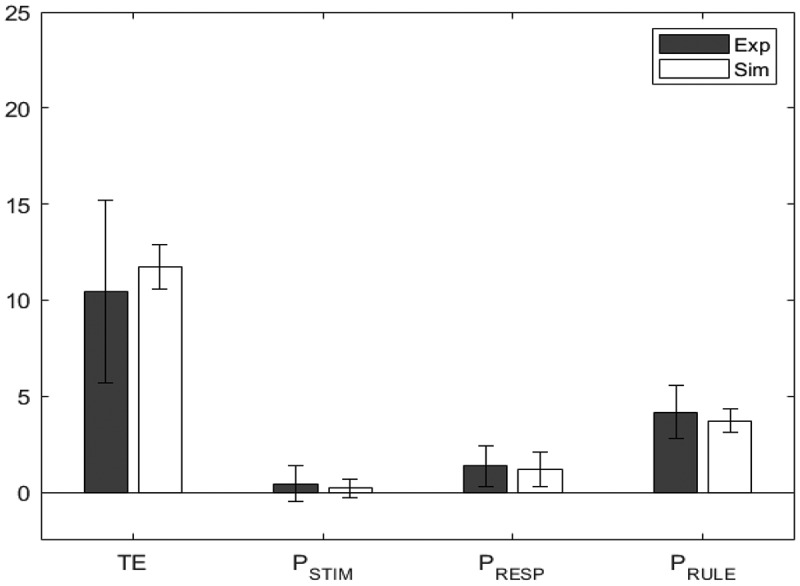
Model fit for younger participants, comparing the experimental (Exp) values in blue/black and the simulated (Sim) values in orange/white, for the BRXT.

**Figure 8.**
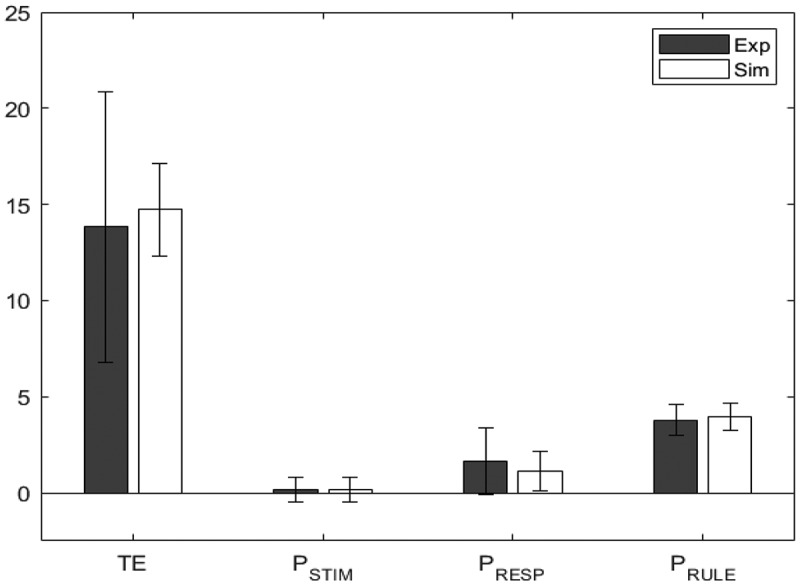
Model fit for older participants, comparing the experimental (Exp) values in blue/black and the simulated (Sim) values in orange/white, for the BRXT.

Table 8 shows the minimized absolute z values for the two groups. As before, a value closer to 0 indicates a better fit. Table 9 shows the resultant values of the parameters of interest for the two groups, while Figure 7 and Figure 8 show bar charts for the dependent measures for the experimental and simulated younger and older participants, respectively. As in the WCST, swapping the age groups worsened the overall fits producing $BF_{01}$ much smaller than 1 for Total Errors (see Table 10). For this task (unlike in the case of WCST) z and $BF_{01}$ values were almost unchanged for the other variables. This is expected, given that TE is the only error whose value differed significantly across the two groups.

**Table 8.** Z statistics for the model fit for the two age groups in the BRXT. A value closer to 0 indicates that experimental and simulated value have closer means, and therefore a better fit. The subscripts near the z value represent the Bayes Factor BF_01_. A value higher than 1 indicates greater support for the null hypothesis, which suggests a good model fit

	TE	$P_{STIM}$	$P_{RESP}$	$P_{RULE}$
*Younger*	$0.073_{1.75}$	$0.069_{2.06}$	$0.024_{3.08}$	$0.102_{1.48}$
*Older*	$0.016_{3.06}$	$0.001_{3.54}$	$0.084_{1.87}$	$0.047_{2.76}$

**Table 9.** Value of the parameters $w_{neg}$, $m_{r}$, ε_str_, and ε_sma_ for the two age groups in the BRXT. Note that for the simulation of the Younger data, m_r_ was fixed at zero and not a free parameter

	$w_{neg}$	$m_{r}$	*ε_str_*	*ε_sma_*
*Younger*	0.162	0.000	0.621	0.512
*Older*	0.048	0.165	0.377	0.089

**Table 10.** Z and Bayes Factor BF_01_ as a subscript for model fits obtained by swapping young and old simulated data. The presence of one BF_s_ below zeros suggest that the two models are not intercheangable and they capture the differences between the two groups

	TE	$P_{STIM}$	$P_{RESP}$	$P_{RULE}$
$Y_{exp},O_{sim}$	$0.814_{0.008}$	$0.093_{1.85}$	$0.054_{2.67}$	$0.021_{3.00}$
$Y_{sim},O_{exp}$	$0.090_{1.45}$	$0.003_{3.48}$	$0.060_{2.20}$	$0.009_{3.36}$

Paralleling the results reported for the WCST model Table 11 shows the number of cycles to selection of a sorting action for the model of the BRXT given the parameter settings determined for younger and older groups, and as a function of feedback on the previous trial. In contrast to the WCST simulations, the BRXT simulations reproduce both the effect of age ($F\left(1,48\right)=15.402$, p<0.001), with simulated older participant group taking more cycles to respond than the simulated younger participant group, and the effect of feedback type ($F\left(1,48\right)=22.403$, p<0.001), with responses following negative feedback taking longer than those following positive feedback. However, and in contrast to the empirical study, the interaction between the factors is significant ($F\left(1,48\right)=11.966$, p=0.001).

**Table 11.** Mean (standard deviation) simulated response time (i.e., number of cycles to generate a response) for each simulated group for trials following positive feedback and trials following negative feedback. Data are based on 25 simulated runs with parameters set as in Table 9

	Positive	Negative
*Younger*	110.1 (1.3)	112.8 (2.7)
*Older*	111.2 (1.4)	128.8 (21.4)

#### Discussion

Whilst the younger group yields an excellent fit when memory for negative feedback ($m_{r}$) is held at 0 and the other three parameters are allowed to vary freely, this is not true for the older group, which in addition requires that memory for negative feedback ($m_{r})$ be greater than 0. Recall that this parameter regulates the extent to which feedback from the previous trial persists to the next trail. Allowing its value to increase when simulating older participants yields an overall model fit that is commensurate with the one observed in the WCST (i.e., with small z→0 and $BF_{01}>1$), and again, inverting the age groups in experimental and simulated data worsens the overall fit, albeit to a lesser extent than in the WCST.^3^ Older participants are simulated with a reduction in the striatal learning rate (*ε_str_*, from 0.621 to 0.337), which corresponds to a slower update of basal ganglia threshold, and a reduction in the sensorimotor schema learning rate (ε_sma_, from 0.512 to 0.089), which corresponds to a sizable decrease in cognitive control. The mismatch reward sensitivity ($w_{neg}$) is also lower for older individuals than younger ones, indicating that, as in the WCST, older participants appear to be more sensitive to negative feedback than their younger counterparts. Fitting the model to older adults also requires a higher value of the memory for negative feedback parameter ($m_{r}$) than the default of 0 for younger participants, suggesting that older participants weigh negative feedback from previous trials more than younger ones.

The simulated RT data may be understood as a prediction of the model based on the best fitting parameter values for the two groups. In this sense, the model fits correctly predict that older participants will take longer than younger participants across all trials, and that responses following negative feedback will be slower than responses following positive feedback for both groups. This is all consistent with the empirical effects, though the magnitude of the RT differences (in model processing cycles) is generally small (of the order of 1 to 2 cycles). It is unclear whether this apparent difference in effect size should be of concern, particularly when, as shown in subsequent simulations, a sizable volume of parameter space yields a good fit (within 1 standard error) to the error data, and when response time following both positive and negative feedback varies substantially in that volume.

### Simulations 3 and 4: model sensitivity and compensatory mechanisms in WCST and BRXT

#### Introduction

Inspection of results during simulated annealing revealed that for both tasks there was often more than one set of parameter values yielding similar z scores. In order to explore the sensitivity of the model’s behavior to the values of the key parameters we ran two additional series of simulations (one for each task) in which the key parameter values were systematically varied across their entire range. Firstly, we expected to find extended regions in parameter space resulting in good fits of the model to the data from each participant group, but a key question of interest was how large such regions might be. Clearly, the answer to this question depends on what is considered a “good fit” to the data. For simplicity, we assume here that a good fit is one where the z score difference between the model and the target participant group is less than 1.00.

A second question addressed by the parameter sensitivity studies concerns the generality of the broad statements relating to the effects of aging and derived from simulations 1 and 2, namely that older participants are better simulated with decreased levels of the three key parameters – mismatch reward sensitivity ($w_{neg}$), striatal learning rate (*ε_str_*) and sensorimotor schema learning rate (*ε_sma_*) – relative to younger participants.

Thirdly, we hypothesized the existence of compensatory processes within the model, whereby variation in one parameter might be approximately countered by a compensatory adjustment in another parameter. More specifically, we hypothesized that an increase in mismatch reward sensitivity ($w_{neg}$) should be compensated by an increase in cognitive control, reflected in an increased striatal learning rate (*ε_str_*) and/or increased sensorimotor schema learning rate (*ε_sma_*) in both tasks, and with comparable model fits.

#### Method

The model was run 25 times (to simulate 25 virtual participants) for each tasks at every point in the multi-dimensional parameter space defined by varying $w_{neg}$, *ε_str_*, and *ε_sma_* from 0.0 to 1.0 in increments of 0.1. For the WCST simulations, this three-dimensional scan was performed twice, once with $m_{r}$ (memory for negative feedback) fixed at 0.0 and once with $m_{r}$ fixed at 0.1. For the BRXT simulations, the three-dimensional parameter space scan was performed four times, once each with $m_{r}$ at 0.0, 0.1, 0.2, and 0.3. For each point in parameter space, the norm of the z-score of the dependent variables (the three error measures for WCST and the four error measures for BRXT) was calculated (i.e., the square root of the sum of squared standard error scores, as in the simulations 1 and 2) with respect to each participant group (i.e., for the data from the younger group and the older group) in order to determine the goodness of fit for each group.

#### Results: WCST

**Figure 9.**
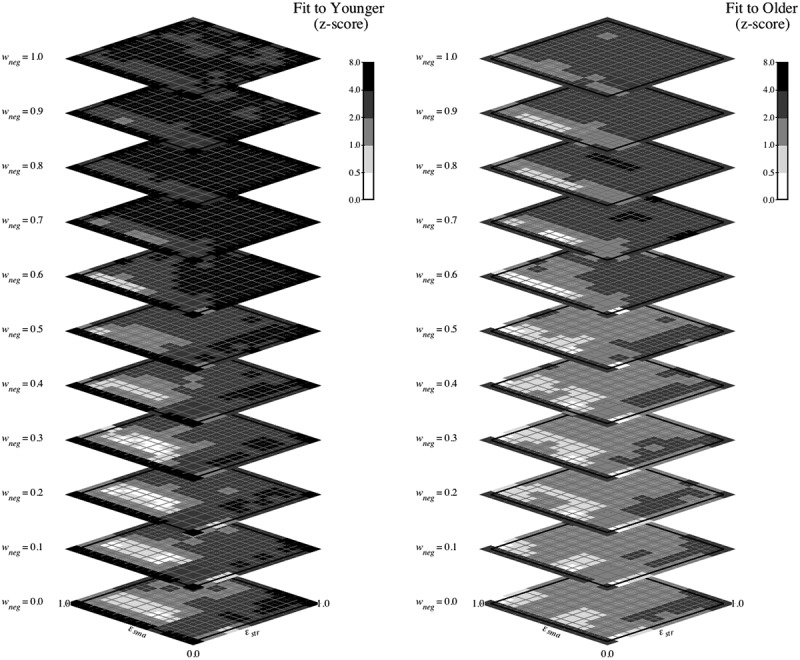
Model fit for younger (left) and older (right) participants, across the 3-dimensional parameter space defined by *w_neg_, ϵ_str_* and *ϵ_sma_* with *m_r_* = 0.0 for the WCST, based on 25 simulated participants at each of 11^3^ points in parameter space. The greyscale shows the z-score fit as described in simulation 1, with red/white representing fits with a *z*-score of less than 0.5 and bluer/darker values representingprogressively poorer fits (i.e., *z* ≥ 0.5).

Figure 9 shows a heatmap representation of the z-score fits of the model configured for the WCST task to the younger (left panel) and older (right panel) data when $m_{r}$ was 0.0. Each panel of the figure shows 11 planes/slices (one for each value of $w_{neg}$ from 0.0 to 1.0 in increments of 0.1), with each plane/slice showing the fit (using logarithmic coding) at each value of *ε_str_* and *ε_sma_*.

The figure shows that good model fits, corresponding to the lighter regions (light gray and white, for z<1), occur over a region of parameter space that extends beyond the fits obtained by simulated annealing in simulation 1. For the younger group, this region extends approximately from values of mismatch reward sensitivity ($w_{neg}$) of 0.0 to 0.4, striatal learning rate (*ε_str_*) of 0.2 to 0.3, and sensorimotor schema learning rate (*ε_sma_*) of 0.4 to 0.9. For the older group, the region of best fit is less easily defined, but extends to higher values of mismatch reward sensitivity ($w_{neg}$ up to 0.9), lower values of striatal learning rate (*ε_str_* down to 0.1), and includes points with zero sensorimotor schema learning (*ε_sma_*= 0.0). Inspection of the corresponding plots when memory for negative feedback ($m_{r}$) was 0.1 revealed a) that there were fewer points of good fit of the model to the younger data, but b) that the fit to the older data was similar for without and with memory for negative feedback ($m_{r}=0.0$ and 0.1, respectively).

#### Results: BRXT

**Figure 10.**
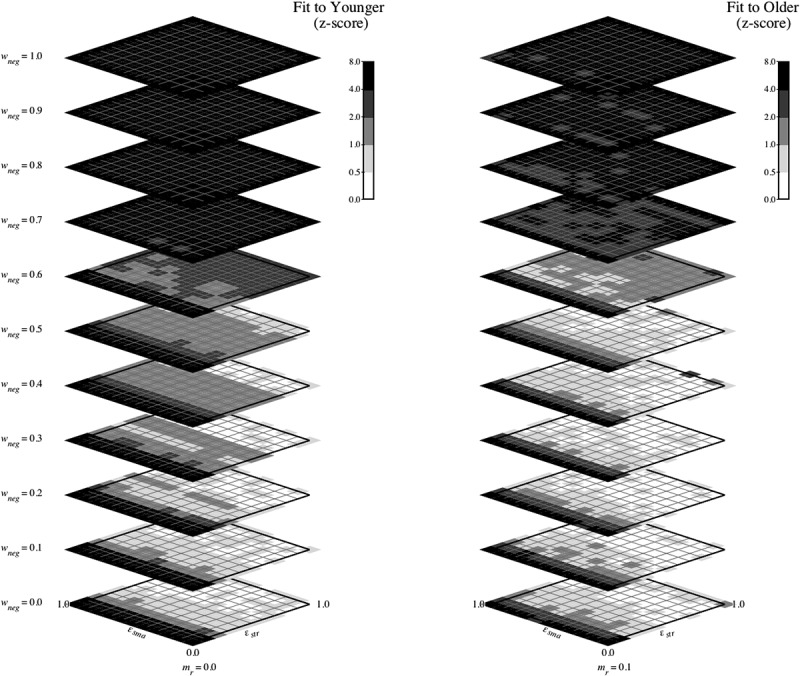
Model fit for younger (left) and older (right) participants, across the 3-dimensional parameter space defined by wneg, *ϵ_str_* and *ϵ_sma_* for the BRXT, based on 25 simulated participants at each of 11^3^ points in parameter space and with *m_r_* = 0.0 for the younger / left panel and *m_r_* = 0.1 for the older / right panel. The greyscale shows the *z*-score fit as described in simulation 2, with white representing fits with a z-score of less than 0.5 and darker values representing progressively poorer fits (i.e., *z* ≥ 0.5).

Figure 10 shows a heatmap representation of the z-score fits of the model configured for the BRXT task to the younger (left panel) and older (right panel) data. As in Figure 9, each panel of the figure shows 11 planes/slices (one for each value of $w_{neg}$ from 0.0 to 1.0 in increments of 0.1), with each plane/slice showing the fit (again using logarithmic coding) at each value of *ε_str_* and *ε_sma_*. Note that the figure only shows data for a subset of the BRXT simulations, namely those with $m_{r}$ yielding the best fits. For the younger panel, $m_{r}=0.0$. For the older panel, $m_{r}=0.1$.

The figure shows that for younger participants good fits (z<1.0) are obtained with memory for negative feedback ($m_{r}$) at 0.0, mismatch reward sensitivity ($w_{neg}$) less than 0.3, and the striatal learning rate (*ε_str_*) greater than 0.3. As mismatch reward sensitivity ($w_{neg}$) increases, the lower limit of the striatal learning rate for this region increases, such that even with moderate mismatch reward sensitivity ($w_{neg}=0.5$) there is a region of good fit when the striatal learning rate is high (*ε_str_*> 0.8). For older participants, good fits are obtained with memory for negative feedback ($m_{r}$) at 0.1, similar values of mismatch reward sensitivity ($w_{neg}<0.6$), and lower values of striatal learning (*ε_str_*> 0.4). In both cases, the model’s fit is relatively unaffected by the sensorimotor schema learning rate (*ε_sma_*).

Inspection of the heatmaps for different values of memory for negative feedback (i.e., $m_{r}>0.0$ for the younger groups and $m_{r}\neq 0.1$ for the older group) reveals in both cases smaller volumes where the fit is good. Thus, the largest volume for the fit to the younger data is obtained with $m_{r}=0.0$ and the largest volume for the fit to the older data is obtained with $m_{r}=0.1$.

#### Discussion

The results of both simulation 3 (for WCST) and simulation 4 (for BRXT) are largely though not entirely consistent with the parameter values determined in simulations 1 and 2, respectively, for each participant group, in that the best fit found through simulated annealing generally falls within a larger region of good fit (informally characterized as z<1) for both participant groups on both tasks. More precisely, on the WCST, for the younger group the point estimate obtained from simulated annealing yielded $w_{neg}=0.457$. The grid-search indicates this produces a fit of z<1.0, but lower values of $w_{neg}$ (e.g., 0.3) yield even better fits (with z<0.5). For the older group, the point estimate determined by simulated annealing is within a region within z=0.5, but this region extends from $w_{neg}=0.0$ to $w_{neg}=0.6$ (with *ε_str_*= 0.1 and *ε_sma_*= 0.0). Interestingly, there is also a region of very good fit with $w_{neg}=0.6$, *ε_str_*= 0.1 and *ε_sma_* ranging from 0.5 to 0.9, indicating that for performance similar to older participants there is a value of mismatch reward sensitivity that makes the model insensitive to the rate of sensorimotor learning when the striatal learning rate is very low. This region also produces a slight RT advantage for trials following positive feedback compared to those following negative feedback, as observed in the empirical data. This local ‘sloppiness’ in the parameter space (Gutenkunst et al., 2007) may be difficult to interpret in this particular region.

For the BRXT, the point estimate for the younger group lies within a region with z<1, but the region extends across the full range of *ε_sma_*, and from $w_{neg}=0.0$, *ε_str_*= 0.3 to $w_{neg}=0.5$, *ε_str_*= 0.9. Similarly, the point estimate for the older group lies within an extended region with z<1.0 (and in fact on the edge of a region with z<0.5). Again, the region is largely independent of *ε_sma_*, and extends from $w_{neg}=0.0$ to $w_{neg}=0.5$. In summary, the grid searches demonstrate that there are extended regions of good fit, more so than might be inferred from the simulated annealing results.

Our second question concerned the generality of the conclusions derived from simulations 1 and 2. Here the results call into question some of our initial findings. The most robust finding is that across both tasks older participants are better characterized by lower values of *ε_str_* than younger participants, which represents a slower update of the basal ganglia threshold, consistent with the previous experiments. There is also some evidence that older participants are better characterized by slightly higher values of memory for negative feedback ($m_{r}$). This is clearest for the BRXT but also apparent from the fit to older participants on the WCST when $m_{r}=0.1$. However, population-level inferences relating to mismatch reward sensitivity ($w_{neg}$) and sensorimotor schema learning (*ε_sma_*) are less robust. Indeed, for both tasks the grid search is suggestive of higher values of mismatch reward sensitivity for older participants than younger ones, while in many cases behavior is not strongly influenced by the sensorimotor schema learning rate.

Our third question concerned the possibility of compensatory interactions between parameter values. The grid searches suggest that in both tasks, decreasing sensitivity of mismatching schemas to feedback (i.e., increasing $w_{neg}$) can be offset by a corresponding increase in the striatal learning rate (*ε_str_*). Effectively, a decrease in mismatch reward sensitivity can be compensated for by an increase in cognitive control. One intriguing possibility is that such compensatory processes may occur in human participants, with aging affecting some variables (e.g., resulting in increased values of mismatch reward sensitivity: $w_{neg}$) but with the effects of that change on performance being ameliorated by a concomitant change in some other variable (e.g., a, possibly deliberate, increase in either striatal *ε_str_* or sensorimotor schema *ε_sma_* learning rates). In theory, such differences might be detectable through neurophysiological measures (e.g., increased *ε_str_* might be mapped onto greater striatal activity, while increased *ε_sma_* might be mapped onto greater Anterior Cingulate Cortex activity). The model therefore predicts that the same behavioral profile on the tasks might be observed with markedly different neurophysiological profiles.

One final issue that requires comment is the difference in parameter values across the two tasks. A naïve application of the model might suggest that, for example, $w_{neg}$ or *ε_str_* should vary with age but not task. However, the optimal values of many if not all of these parameters are likely to be dependent on specific aspects of a task, such as the number of schemas (assumed to be three cognitive and four sensorimotor in WCST, and five cognitive and nine sensorimotor in BRXT) and the form of feedback (which is explicit in WCST, but implicit in BRXT). Differences across tasks in the values of these parameters should therefore not be surprising.

## General discussion

In the present paper, we have reported the results of a study in which we tested 25 young adults and 25 adults over the age of 60 who completed a variation of the Wisconsin Card Sorting Test and a variation of the Brixton Spatial Anticipation Test. We predicted that in the WCST we would observe an increase in set loss errors in older adults, without a significant change in perseverative errors. In BRXT we predicted that we would observe more total errors in older participants than younger participants, but not more perseverative errors. We furthermore predicted that, across all participants, the number of total errors on the two tasks would correlate, and that the number of perseverative errors made on each task would correlate. Each of these predictions was born out, with one notable caveat: the number of perseverative errors made in the WCST was found to correlate with the number of response perseverations and the number of stimulus perseverations made in the BRXT, but not the number of BRXT rule perseverations.

That older participants were more susceptible than their younger counterparts to set loss errors, but not perseverative errors, on the WCST supports the view that these errors have different origins. The former reflect failure to maintain set in the face of interference, while the latter reflect failure to switch set in the face of negative feedback. Both tendencies are frequently reported in frontal patients (see, e.g., Shallice, 1988 ch.14). Within the model, the dissociation occurs because set loss errors primarily occur with reduced mismatch reward sensitivity (when $w_{neg}$ is high) or slowed sensorimotor schema learning (*ε_sma_* is low), while perseveration errors primarily occur when striatal learning is slow (*ε_str_* is low) but sensorimotor learning is fast (*ε_sma_* is high).

With regard to the BRXT, while both groups of participants produced a similar number of perseverative errors, older participants produced more total errors than younger participants. Total errors include ones which might be conceptualized as equivalent to set loss errors on the WCST (i.e., switching away from an established rule despite positive feedback), as well as errors related to rule induction (i.e., deriving a new rule on the basis of patterns in the temporal input). Indeed, within a neuropsychological context, Reverberi et al. (2005) argue that the BRXT is primarily a task of rule induction rather than of rule switching. While our results do not differentiate the possible causes of non-perseverative errors, they are consistent with the view that the BRXT does not specifically assess tendencies toward perseveration. Within our model, rule induction consists in attempting all known rules that are consistent with recent feedback. The model produces more total errors (but not more perseverative errors) when memory for negative feedback ($m_{r}$) is relatively high, the striatal learning rate (*ε_str_*) is above 0.3, and mismatch reward sensitivity ($w_{neg}$) is not more than 0.5. It appears that when feedback persists over multiple trials (as when $m_{r}$ is greater than zero) the model is less effective at inducing rules.

While the BRXT was originally developed as a test of frontal dysfunction (Burgess & Shallice, 1996), more recent neuropsychological evidence has called into question the test’s structural specificity. In particular, Mole, Foddai, Chan, Tianbo, & Cipolotti (2020) found that the original BRXT was no more sensitive to frontal lesions than posterior ones, while Vordenberg, Barrett, Doninger, Contardo, & Ozoude (2014) found that stroke patients with subcortical lesions performed more poorly on the task than those with frontal lesions. Our results provide a way of making sense of these more recent findings, in that while performance of the model on the BRXT is relatively insensitive to *ε_sma_*, a parameter related to cortical learning, it is not insensitive to *ε_str_*, which determines subcortical learning. In other words, the model’s behavior is consistent both with the findings of Vordenberg et al. (2014) and Mole et al. (2020).

A further important empirical result was the observed correlation between PE on the WCST and both $P_{RESP}$ and $P_{STIM}$, but not $P_{RULE}$, on the BRXT. It is widely accepted in the neuropsychological literature that there are different forms of perseveration, and that these are associated with distinct neural sites (e.g., Sandson & Albert, 1984). One strength of the BRXT, even if conceptualized as a test of rule induction rather than perseveration, is that it allows one to discriminate between different forms of perseveration. The observed cross-task correlations suggest that, even in a non-clinical sample, there are individual differences in the tendency to produce stimulus-driven or response-driven perseverations, and that these forms of perseveration are in large part responsible for the perseverative errors observed in non-clinical populations on the WCST. The separate levels of schemas within our model – sensorimotor schemas and cognitive schemas – provide an obvious locus for explaining the observed correlation. Suboptimal regulation of sensorimotor schemas, and in particular failure of top-down regulation of such schemas, should lead to both increased levels of PE on the WCST and increased levels of $P_{RESP}$ and $P_{STIM}$, but not $P_{RULE}$, on the BRXT. However, the limited evidence for the null hypothesis in the $PE/P_{RULE}$ correlation, as evinced by a low Bayes factor ($BF_{01}=1.53$), does not allow us to draw a strong conclusion regarding the relationships between perseverations. Nevertheless, there is suggestive evidence supporting a non-unitary and hierarchical view of perseveration.

In addition to the above, for both tasks we found that older participants were slower to respond than younger participants. This is not surprising and is consistent with a substantial body of previous literature (see Cerella & Hale, 1994 for a review). In both groups, there was also an increase in response time on those trials that followed negative feedback as compared to those that followed positive feedback. Again, this should not be surprising as the former case requires additional cognitive processes – inferring a new potential rule and reconfiguring cognitive processing to follow that rule – beyond those required in the latter case. It is not clear whether the two factors interact. Across both tasks, the increase in response time following negative as opposed to positive feedback was slightly, although non-significantly, greater in the response times of older participants than younger participants. An additive effect would suggest that the time taken for the additional processing required following negative feedback was independent of age, while a super-additive effect would be consistent with generalized slowing of cognitive and motor processing with age. Our results (particularly with the WCST) are suggestive of the second of these possibilities, though consistent with both possibilities.

At the same time, the least satisfactory element of our model is the apparent insensitivity of response times on trial n to feedback on trial n−1, at least as reported in the results of simulations 1 and 2 above. This might be attributed to the lack of complex reasoning processes that might be recruited for rule induction within our model (such as those discussed with respect to the WCST by Dehaene & Changeux, 1991). Alternatively, from the predictive processing perspective of (Barceló, 2020) described above, it may be attributed to differences arising from successful versus unsuccessful prediction. However, it should also be noted that for some regions in the parameter space the model does produce reliable (and statistically significant) differences in response times as a function of prior feedback. Moreover, as reported in our previous work (Caso & Cooper, 2020), the model captures the different cortical responses elicited during early (rule induction) and late (rule application) trials along each card sorting series, as observed in electrophysiological studies (e.g., Barceló, 2003). This suggests that RT effects might be used as an additional constraint on parameter selection.

Neurobiologically, aging has been associated with a decreased concentration of neurotransmitters such as dopamine and noradrenaline in both frontal and striatal circuits (see, e.g., Kaasinen & Rinne, 2002). Our modeling results are consistent with this. More specifically, the modeling provides evidence for the decline of two domain-general mechanisms in the older group, characterized in the model as *ε_sma_* and *ε_str_*, and at a more mechanistic level as cognitive control and basal ganglia update. Findings from the modeling with respect to the $m_{r}$ parameter further suggest that aging might also affect the extent to which feedback from the previous trial affects the processing of the current one. This may represent a measure of feedback perseverance or inertia.

One intriguing possibility in the context of the aging brain is that the reparameterization necessary to simulate the behavior of the older group with respect to the younger group can be understood as a product of compensatory mechanisms. In particular, older participants might compensate for reduced mismatch reward sensitivity (higher $w_{neg}$), but also greater feedback persistence (greater $m_{r}$), by increasing cognitive control (cortically or subcortically, via greater *ε_sma_* or *ε_str_* respectively). This possibility is consistent with the idea that the engagement of neural circuits in cortical structures is higher for older adults when the task load is lower, either because resources are not efficiently deployed, or alternatively because input in the prefrontal cortex is degraded (perhaps because of insufficient neurotransmitter concentration), as suggested by Reuter-Lorenz & Cappell (2008).

## Conclusion

We have presented data which demonstrates that older participants tend to err more on the WCST and BRXT than their younger counterparts, but that the increased rates of errors of older participants are not due to perseverative rule application. Moreover, our data demonstrate that, across the age spectrum, the tendency to perseverate on the WCST is positively associated with the tendency to produce stimulus and response (but not rule) perseverations on the BRXT. At a cognitive level, the increased rates of errors of older participants appear to be the product of reduced effectiveness of proactive control, rather than of decreased effectiveness of reactive control. Our neurobiological model demonstrates that both tasks can be conceptualized as modulated schema-driven behavior, and allows us to relate the cognitive-level effects to the neural level. Specifically, the model suggests that the decreased effectiveness of proactive control is in part or whole due to weaker adaptation during the tasks within the striatum, particularly when integrating the consequences of negative feedback for mismatching response schemas.

## Notes

1. For complete details, see (Caso and Cooper, 2020), and for complete source code for the simulations reported there, see https://github.com/AndreaCaso/wcst-pd.

2. In the experimental section of the paper, we reported $BF_{10}$ as a measure of the likelihood of the alternate hypothesis relative to the likelihood of the null hypothesis. Here we report its reciprocal ($BF_{01}$), as it is more appropriate as an index of model fit.

3. This is consistent with the differences between groups across the two tasks, i.e., that the difference between younger and older participants is less on the BRXT than on the WCST.
